# A brief pre-visit educational video improved patient engagement after telehealth visits; results from a randomized controlled trial

**DOI:** 10.1016/j.pecinn.2022.100080

**Published:** 2022-09-05

**Authors:** Howard S. Gordon, Oksana Pugach, Pooja Solanki, Ravi K. Gopal

**Affiliations:** a Jesse Brown Veterans Affairs Medical Center and VA Center of Innovation for Complex Chronic Healthcare, Chicago, IL, USA; b Section of Academic Internal Medicine, Department of Medicine, University of Illinois at Chicago, Chicago, IL, USA; c Institute for Health Research and Policy, University of Illinois at Chicago, Chicago, IL, USA; d Center of Innovation for Veteran-Centered & Value-Driven Care, Rocky Mountain Regional Veterans Affairs Medical Center, Aurora, CO, USA; e Department of Medicine, Division of General Internal Medicine, University of Colorado School of Medicine, Aurora, CO, USA

**Keywords:** Video Telehealth, Patient-centered care, Randomized Trials, Veterans, Physician-patient communication

## Abstract

**Objective:**

To test an intervention designed to improve patient engagement in telehealth visits by encouraging patients to use active communication behaviors.

**Methods:**

US Veterans with type 2 diabetes mellitus receiving primary care using telehealth were randomized 1:1 to receive both a pre-visit educational video and pamphlet (intervention) or pamphlet alone (control) prior to their scheduled telehealth visit. Data were collected before and after the intervention from the medical record and at telephone interviews (questionnaires). Analyses compared the intervention and control groups using bivariate statistics and multiple regression.

**Results:**

There were no statistically significant differences in baseline Hemoglobin A1c (HbA1c) between intervention and control groups (*P* > 0.05). Patient's ratings of physicians' communication and post-visit empathy were higher (*P* ≤ 0.05) in the intervention group than control group and after adjusting for baseline values the intervention group reported higher scores on post-visit therapeutic alliance with the provider and higher patient engagement, compared with the control group, *P* = 0.01 and *P* = 0.04, respectively, but post-visit HbA1c was not statistically different.

**Conclusions:**

The educational video was useful as pre-visit preparation for patients prior to a primary care telehealth visit.

**Innovation:**

This study showed the efficacy of a pre-visit video to improve patient engagement and therapeutic alliance after telehealth visits.

**ClinicalTrials.gov****Identifier**: NCT02522494

## Highlights

• The study tested a pre-visit intervention designed to improve patients' communication in a telehealth visit.
• The video + pamphlet improved patients' ratings of therapeutic alliance and engagement compared with getting the pamphlet alone.
• A video-based pre-visit intervention may be effective at improving visit outcomes in video telehealth visits.

## 1 Introduction

Video telehealth medical care visits have increased in use and were widely adopted by both patients and providers especially in the era of physical distancing during the COVID-19 pandemic. However, telehealth encounters involve remote communication that precludes traditional greetings such as a handshake, limits the participants observation of each other, does not allow hands on evaluation, and may present technology challenges for patient and provider. Because of these and other differences between telehealth and in-person visits, patient-provider communication during telehealth visits may be less patient-centered and may contribute less to the development of a therapeutic provider-patient relationship when compared with communication during in-person visits [[1], [2], [3]].

Given the increased use of telehealth visits, efforts to improve the patient experience in these visits is important because improving the patient experience improves the quality of healthcare [[4], [5], [6], [7], [8], [9]]. One way to improve the patient experience is to make healthcare more patient-centered by increasing patients involvement in their care. Patient-centered care depends on clinicians to identify patients' values and preferences and on patients to be prepared to be actively engaged in their visits [[10], [11], [12]]. Patients who are actively engaged in their visits gain more control over their care by communicating with their provider about their needs, concerns, and questions. When patients use active communication behaviors, providers generally respond in kind because of norms of communication (e.g., questions lead to answers). Yet coaching interventions to encourage active patient involvement in their medical visits have received limited attention. Studies of provider-patient communication that use interventions to improve patients' communication behaviors have had more success at enhancing the provider-patient encounter than those attempting to improve providers' communication behaviors [13,14]. Furthermore, patients' active participatory communication behaviors are associated with positive outcomes, including adherence to and recall of providers' recommendations, patient satisfaction, improved functional status, and even improved biomedical or physiological outcomes [14].

Interventions that encourage patients to use active communication behaviors in face-to-face visits used coaches, paper-based methods [[15], [16], [17]], or “web-based” tools [[18], [19], [20]]. These interventions had modest effects on patient communication behaviors and have not been routinely adopted in practice, perhaps due to the cost of trained personnel to deliver the coaching, or insufficient level of patients' health or computer literacy for workbooks or web tools. Though there are tools to improve provider-patient communication in telehealth visits, most focus on improving provider's “webside manner” [21]. Only a few studies have examined video-based interventions to improve communication in medical in-person interactions [[21], [22], [23], [24]].

In this study, we tested the efficacy of a pre-visit educational video for patients, titled “Speak Up,” that encourages patients to use active communication behaviors and to speak up during the visit. A video-based intervention offers several advantages for patient education over other approaches. Video-based education is acceptable to patients from a broad range of cultural backgrounds [25], and video may be more easily disseminated than interventions requiring trained coaches.

## 2 Methods

### 2.1 Patients

Eligible patients with a diagnosis of type 2 diabetes mellitus, a HbA1c ≥ 7%, and who received primary care with clinical video telehealth (CVT) were identified with a search of electronic health records. Patients were mailed an invitation to participate and those who agreed were enrolled in a randomized controlled trial to test the efficacy of the pre-visit educational video. Patients were United States Veterans, lived in rural communities, and utilized care from clinical video telehealth clinics in two large US Department of Veterans Affairs (VA) health networks in the central United States. Patients' residences were geographically distant from the providers' locations at urban VA medical centers. Patients attended a telehealth clinic at a local community-based outpatient clinic and communicated with the provider through the telehealth video and audio communication technology. Patients were screened for dementia and hearing loss that would have prevented them from participating in the telephone interview. Providers who saw eligible patients were invited to complete a demographic questionnaire, were mailed the provider pamphlet (see below), and were informed that patients with diabetes were invited to enroll in the study. Providers were not informed whether patients agreed to participate in the study. The study was approved by the VA Central IRB (#14–22) and all patients provided verbal informed consent by telephone to participate.

### 2.2 Design and enrollment

This study was a randomized controlled trial of a patient education video developed for this study. Participants were randomized to receive the “*Speak Up!*” video and a pamphlet (intervention arm) or the pamphlet alone (control arm). *Speak Up!* was designed to encourage patients to use active participatory communication behaviors in telehealth visits. The video is 12 minutes in length, has a friendly narrator and shows actor-patients role modeling and overcoming common communication challenges in telehealth visits. Viewer engagement was enlisted by including content in the video from previous qualitative research with VA patients with diabetes [2,26]. Content was designed to engage viewer attention with humor, multimedia, and to dispel several myths patients described about communicating with their providers [26]. Actors were selected to show a diverse group of patients by sex and gender, so viewers might be more likely to be attentive and to self-identify with the scenarios portrayed. In the video, three actor-patients (a black male, Hispanic female, and a white male accompanied by his wife) with diabetes, an actor-nurse, and an actor-physician portray scenes from the waiting room, nurse check-in, and exam room in a simulated clinical video telehealth clinic. The video was filmed at the Graham Clinical Performance Center (GCPC) at the University of Illinois Chicago. GCPC has equipment that can realistically depict telehealth visits.

An expert panel consisting of providers, public health experts, and doctoral-level communication experts developed the video by integrating input from previous qualitative research with patients [2,26], and providers [3], and from frameworks of communication competence and social learning theory [27,28]. A similar video we developed for in-person visits was evaluated for acceptability and feasibility with 10 in-depth interviews with patients with diabetes and in 50 patients at new patient orientation meetings. Patients who watched the video reported improved understanding of their role with the healthcare provider, reported they were more likely to ask questions and reported they were more likely to share health information and concerns with the provider. The video was scripted to incorporate the importance of observing and modeling the behaviors, attitudes, and reactions of others when learning new behaviors [28]. The script was written to dispel myths and use humor to address common barriers to communication and encourages preparation for the visit and the use of active communication behaviors such as asking questions, making requests, and expressing concerns or opinions. The video encourages viewers to prepare for their visit and includes reminders about diabetes self-management and medication adherence. For example, the nurse encourages one patient to speak up to her provider because of her concerns about insulin and weight gain. Another patient who forgets what he wants to say receives suggestions about being prepared by making a list and instructions for how to reach the doctor when he remembers. The full video is available at https://bcove.video/31knTfg.

Similar to the video, the pamphlet (reading grade level 5) was developed for the study and describes how to use active communication behaviors in a telehealth visit (Supplemental Fig. 1) and was based on a model of active patient participation [29]. The intervention materials (DVD and pamphlet) were mailed to participants randomized to the intervention arm. An internet link to the video was also provided. If patients did not have access to a DVD player, a DVD player was mailed to them (*n* = 4). Participants in the control arm received the pamphlet by mail. We created another pamphlet as a guide for providers and CVT personnel to encourage active patient communication in telehealth visits (supplemental fig. 2). The provider pamphlet was distributed by mail and e-mail and was developed by our expert panel based on qualitative analysis of provider interviews and the Four Habits model [3,30].

### 2.3 Power calculation, randomization, and recruitment

The study was designed with a target power of 80%, an effect size of 0.40 and a one-sided alpha = 0.05 (for an intervention designed to improve HbA1c, there was no possibility of claiming significance of the opposite result) and targeted enrolling a sample size of 80 subjects. We used restricted block randomization with equal allocation across two groups. The stratification was by provider using blocks of various sizes, size 2 and 4. The random allocation table was generated using random number generation functions in SAS v. 9.2 and was loaded to the REDCap randomization module by the statistician.

**Fig. 1:**
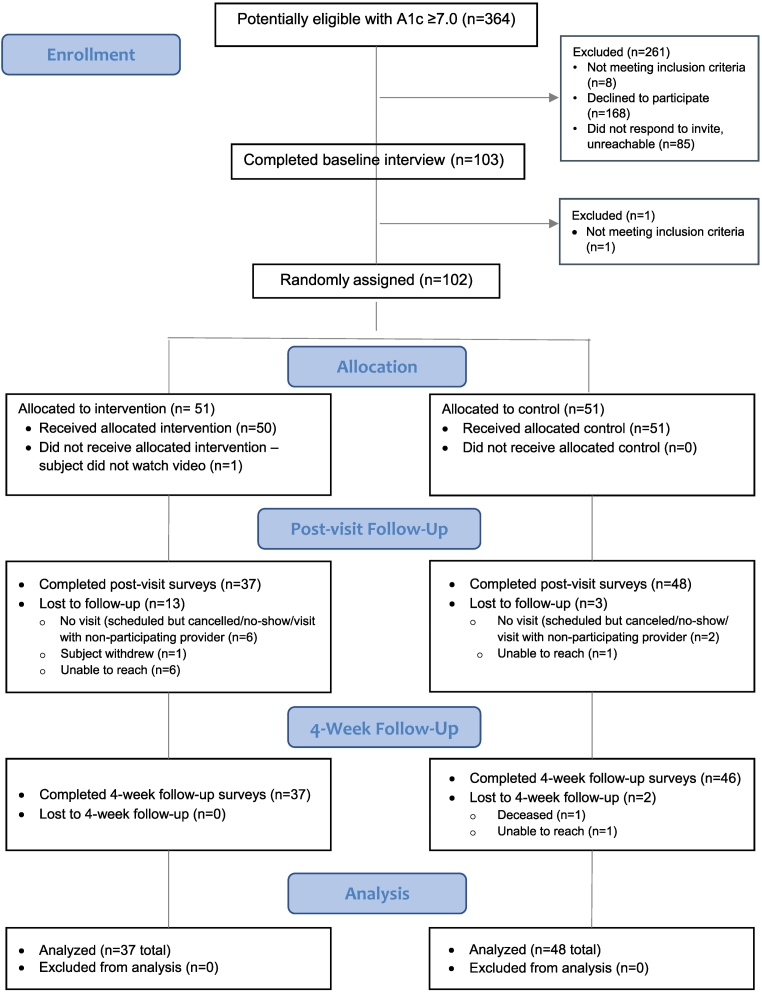
CONSORT Diagram.

We identified 364 potentially eligible patients, enrolled 103, of which 102 met the inclusion criteria and were randomized by research staff unaware of the random sequence prior to randomization. Patients were randomized 1:1 to receive both the Speak Up video DVD and patient pamphlet (intervention arm) or the pamphlet alone (control arm) by US first class or overnight mail 7–21 days prior to their scheduled video visit. All randomized patients received the allocated intervention or control. Patients completed pre-visit and post-visit telephone interviews to collect data on demographics, covariates, and outcomes. Because the intervention was mailed there was no cross-over between the two study arms. 85 patients completed the study (CONSORT Fig. 1).

### 2.4 Data and measurement

Data were collected by self-report on demographics and survey measures at baseline including health literacy, social support, depression screen, and functional status [[31], [32], [33]]. We evaluated the delivery of the intervention video and the pamphlet with a 5-item Intensity and Fidelity Survey several days before the telehealth visit, with the highest possible score of 5 reflecting the highest level of engagement [34]. Within 7 days after the telehealth visit we conducted a post-visit telephone interview to collect patients' ratings of communication, self-efficacy, trust, relational empathy, and patient centered care [[35], [36], [37], [38], [39]]. Adherence was measured at a 4 week post-visit follow-up telephone interview using the medical outcomes study measure (MOS) and the resistance to treatment survey. Resistance to treatment is a 20-item instrument that identifies reasons for non-adherence by assessing emotional and physical factors related to the patient's treatment and care for diabetes [40]. MOS is a five-item questionnaire that measures patient adherence to physicians' recommendations [41]. In addition to adherence, patient satisfaction with their healthcare and their provider were assessed at the 4-week follow-up interview.

We collected and compared pre-visit and post-visit ratings of patients' self-efficacy to communicate using the 5-item PEPPI measure [42], trust in physician and trust in health care [43], diabetes self-efficacy [44], patient engagement [45], shared decision making [46], and therapeutic alliance[47] between the intervention and control groups. We measured therapeutic alliance with the provider with the Human Connection Scale modified by substituting “diabetes” for “cancer” in the question “How often does your doctor ask how you are coping with cancer?” This 15-item scale assesses mutual caring, respect and understanding [47]. Patient engagement was assessed using 7-items from the Altarum Consumer Engagement survey (ACE), which is predictive of health status, lifestyle behaviors, and medication adherence [45]. Patients' ratings for each measure were normalized to a 0–100 scale. Hemoglobin A1c results (primary outcome) were obtained pragmatically from the electronic medical record at baseline and a median of 192 days (IQR 112 to 267 days) after the visit. Hemoglobin A1c was chosen as the primary outcome to provide an objective clinical outcome. Recruitment was completed in March 2020.

### 2.5 Analysis

The analytic sample included participants with data at both pre- and post-visit (per protocol). An intention to treat analysis of the A1c outcome was not different than the per protocol analysis. Descriptive bivariate statistics were calculated with *t*-tests or chi square tests as appropriate for patient demographics, survey measures, and outcomes. Analyses compared the intervention and control groups using multiple regression to control for baseline measures. We considered a two-sided *p*-value of 0.05 as significant. All analyses were conducted using SAS 9.4.

## 3 Results

A total of 85 patients were included in this study (Table 1). Most of the sample was male (*n* = 83) and white (*n* = 73). The mean age was 65 years. There were no statistically or clinically significant differences in age, gender, race, education, income, social support, depression screen, and physical or mental functional status between intervention and control groups (*P* > 0.05; Table 1).

**Table 1:** Baseline patient demographics and clinical characteristics (*N* = 85).

Baseline demographics	Control (*N* = 48) Percent (N) or Mean (SD)	Intervention (*N* = 37) Percent (N) or Mean (SD)	P-value
Age (mean years, SD)	64.8 (9.4)	65.5 (8.4)	0.75
Male	95.8% (46)	100% (37)	0.50
White (*n* = 81)	93.3% (42)	86.1% (31)	0.46
Hispanic (n = 83)	34.0% (16)	36.1% (13)	0.84
High School Graduate	93.8% (45)	97.3% (36)	0.63
Married	60.4% (29)	78.4% (29)	0.08
Social support ^a^ (mean, SD)	14.1 (7.2)	16.1 (6.6)	0.21
Unemployed/retired	80.9% (38)	73.0% (27)	0.39
Live with someone	81.3% (39)	83.8% (31)	0.76
Income >20,000 (*n* = 82)	84.8% (39)	88.9% (32)	0.75
Study Sites			
Site 1	93.8% (45)	91.9% (34)	1.00
Site 2	6.2% (3)	8.1% (3)	
Baseline characteristics			
Health literacy (mean, SD)^b^	3.4 (1.4)	3.5 (1.3)	0.75
Depression ^c^	2.1 (1.8)	2.3 (2.1)	0.72
Peripheral Neuropathy ^d^	9.6 (4.3)	10.9 (4.4)	0.17
Mental functional status ^e^	49.3(24.6)	45.9 (22.9)	0.51
Physical functional status ^e^	35.8 (21.2)	34.9 (21.1)	0.84

Measures had possible range from 1 to 5 for health literacy, ^a^ from 4 to 28 for social support, ^b^ from 2 to 8 for Depression, ^c^ from 4 to 20 for Peripheral Neuropathy, ^d^ and from 0 to 100 for mental and physical functional status summary scores, ^e^ with higher scores signifying higher health literacy, higher social support, more likely to be depressed, worse symptoms of peripheral neuropathy, and higher mental and physical and functioning scores, respectively.

In the intervention and control groups, there was no significant difference in the proportion of participants who read the pamphlet. In the intervention group, participants reported their level of engagement when watching the video as very attentive (46%), attentive (27%), moderately attentive (8%), slightly attentive (8%), and inattentive or did not watch the video (11%).

Patient ratings of trust, communication self-efficacy, patient engagement, shared decision-making, provider-patient therapeutic relationship and HbA1c did not significantly differ when examined in separate pre-visit and post-visit bivariate comparisons (Table 2).

**Table 2:** Outcomes measures at pre- and post-intervention in control vs. intervention (n = 85).

	Control (N = 48) Mean (SD†)	Intervention (N = 37) Mean (SD)	P-value
Patients' ratings of: * Trust in provider or physician			
Pre	80.2 (21.1)	83.8 (21.6)	0.49
Post	82.2 (23.3)	86.3 (15.6)	0.37
Trust in VA healthcare system			
Pre	68.5 (25.3)	70.9 (24.2)	0.66
Post	68.8 (26.2)	77.1 (21.6)	0.12
Self-efficacy to communicate			
Pre	70.6 (28.9)	72.2 (26.8)	0.80
Post	77.8 (21.7)	83.5 (20.2)	0.22
Patient engagement			
Pre	69.6 (20.0)	68.6 (14.9)	0.81
Post	71.7 (20.0)	77.0 (15.4)	0.20
Participatory decision-making style			
Pre	72.8 (31.4)	79.3 (29.1)	0.38
Post	69.8 (32.2)	83.1 (29.8)	0.06
Diabetes self-efficacy			
Pre	73.5 (24.8)	78.4 (19.2)	0.33
Post	78.1 (24.5)	78.9 (19.8)	0.87
Therapeutic alliance			
Pre	79.3 (23.6)	81.9 (18.9)	0.61
Post	79.5 (21.8)	86.9 (15.1)	0.07
HbA1c‡ (%, SD)			
Pre	8.5 (1.20)	8.5 (1.76)	0.95
Post	8.2 (1.26)	7.99 (1.28)	0.34

* Measures are normalized to have a possible range from 0 to 100.

† SD denotes standard deviation

^‡^ HbA1c denotes hemoglobin A1c

Compared with controls, patients in the intervention group rated their provider as more informative and reported they understood the information to a greater extent (88.8 vs 80.7%; *P* = 0.04), gave higher ratings to overall communication (89.4 vs. 82.4%; *P* = 0.05), and patients gave higher ratings of providers empathy (86.6 vs 76.0%; P = 0.04), but there was no significant difference (*P* > 0.05) in satisfaction, patient-centered care, and adherence to treatment (Table 3).

**Table 3:** Post-visit patient survey measures in control vs. intervention (N = 85).

Survey measures	Control (N = 48) Mean (SD)*	Intervention (N = 37) Mean (SD)*	P-value
Health rating	57.6 (26.2)	56.8 (19.4)	0.87
Communication ratings	82.4 (20.2)	89.4 (12.0)	**0.05**
Doctor's Informativeness	80.7 (21.4)	88.8 (13.8)	**0.04**
Patient's Participation	83.9 (23.8)	89.4 (16.4)	0.21
Doctor's Supportiveness	82.6 (22.9)	90.5 (17.1)	0.08
Read the pamphlet [Percent (N)]	91.7% (44)	89.2% (33)	0.72
Watched the video [Percent (N)]	n/a	89.2% (33)	n/a
Satisfaction with healthcare	68.1 (28.9)	74.3 (23.8)	0.30
Satisfaction with doctor	84.8 (24.0)	87.4 (81.9)	0.56
Relational empathy	76.0 (26.8)	86.6 (19.4)	**0.04**
Patient centered care	76.6 (25.1)	83.4 (17.8)	0.16
Adherence to treatment	68.6 (17.5)	71.8 (13.8)	0.37
Adherence to physicians' recommendations	76.0 (26.4)	78.5 (21.5)	0.64

* Except where noted values are mean and standard deviation. Measures had possible range from 0 to 100.

To evaluate whether there were significant improvements in several measures from before to after the intervention, we conducted regression analyses to adjust outcomes shown in Table 2 for baseline measures. After adjusting for baseline measures there remained no statistically significant difference in post-visit self-efficacy to communicate, trust in physician, or HbA1c between intervention and control groups (*P* > 0.05). However, controlling for baseline measures, ratings of post-visit therapeutic alliance with the provider and ratings of patient engagement were higher in intervention group patients [8.6% (SE 3.3); *P* = 0.01; and 6.6% (SE 3.2) *P* = 0.04; respectively] compared with patients in the control group.

## 4 Discussion and conclusion

### 4.1 Discussion

In this study, we conducted a randomized controlled trial of a mailed pre-visit educational intervention in patients with type 2 diabetes scheduled for telehealth visits. We found statistically significant and higher patients' ratings of perceived level of physicians' informativeness, overall communication and empathy in the intervention compared with the control arm and we found that patients reported higher levels of therapeutic alliance with the provider and higher engagement in their care. However, no statistically significant differences were found in patients' post-visit ratings of self-efficacy to communicate, shared decision-making, patient-centered care, or patients' satisfaction in intervention versus control group. These results are important because they demonstrate that a brief pre-visit video that encourages patients to actively communicate during their visit appears to be effective at improving several communication-related outcomes in a telehealth visit. The results suggest that despite reported difficulties with developing a provider-patient relationship in telehealth visits [2,48,49], an educational pre-visit video might help patients prepare for their visits and may reduce communication barriers, thereby improving visit outcomes.

While telehealth has been promoted as effective means of providing health services, especially during the COVID-19 pandemic, many of the studies that have explored patients' experiences in telehealth visits were focused on patients' satisfaction [[50], [51], [52], [53]]. Previous studies have shown that patients were satisfied with telehealth visits with respect to travel times and improved access to appointments or the studies compared ratings of communication in telehealth to in-person visits. Yet, there are fundamental differences in communication in telehealth visits compared to in-person medical encounters. The diminished humanistic features of a telehealth visit could impair provider-patient communication, which may impede the development of the provider-patient relationship and reduce patient's trust or adherence to the provider's recommendations. Several studies have shown that activating patients to use more effective communication methods with their provider can improve health outcomes [54,55]. In our prior study, patients with diabetes identified communication issues they experienced in telehealth visits [2]. Using those results we developed a pre-visit video intervention “Speak Up” for telehealth that provides an opportunity for patients to view and model acceptable, positive, and powerful active communication behaviors. Our video offers pre-visit preparation that otherwise would require trained staff and based on our results may be better than paper-based educational materials. Prior studies have also noted limited effectiveness of pamphlet or workbook materials for patient education [16,56]. To our knowledge, this study is the first to examine how a pre-visit educational video could improve patients' engagement in telehealth visits.

This study adds to the medical literature on communication during telehealth visits because of its unique design. The study intervention was designed to improve patient's communication behaviors. Encouraging active patient communication supports the patient role in the medical interaction and assists providers' efforts to obtain their patients' history and lead a shared decision-making discussion and may improve patient care [26,57]. The results of our study also add to the literature that paper-based interventions such as the pamphlet used in the control arm are insufficient to promote patient's engagement in medical visits [58].

In our study, we did not find statistically significant differences in patients' post-visit ratings of communication, shared decision-making, patient-centered care, patient satisfaction, or self-efficacy to communicate in intervention vs. control group. One of the possible explanations for this finding may be related to patients' interpretation of their own communication behavior. It is possible that patients were not able to accurately provide self-assessments and thus gave overestimates of their performance, which may be more common in self-assessment of interpersonal skills [59]. Future studies may attempt to overcome this possibility by having independent observers rate recordings of the interactions. Independent external observers may have a different perspective because they are not involved in the interaction. Research has shown that patients' ratings of their own active communication behaviors or their ratings of physicians' participatory decision making style are not correlated with independent observers' ratings of those behaviors [60]. Another possible explanation for this insignificant finding could be the varying levels of engagement while watching the intervention video. Participants were not fully attentive while watching the DVD. When we assessed by intensity and fidelity of the intervention by telephone survey before the telehealth visit, 25% of the patients in the intervention group did not remember specifics about the actor-patients in the video. We did not find demographic or other factors associated with attentiveness to watching the video.

This study has several limitations. First, the sample size was small and limited by the relatively low availability of CVT visits conducted by primary care providers in the period prior to the COVID-19 pandemic. Second, our study population consisted of US Veterans, mostly male, white, and from two geographic areas, and thus, our results may not be generalizable to other population groups, women, or other health care systems. Third, we developed and delivered an educational pamphlet to providers as an aid to encourage patients' communication. Although the provider pamphlet might have improved providers' communication, our study was not designed to evaluate providers' communication. Fourth, A1c measurements were completed at a large range of time points post-visit perhaps limiting our ability to detect a change in A1c. However, these limitations are balanced by several strengths including the pragmatic design to implement delivery of the study intervention prior to scheduled telehealth visits and to collect A1c according to the standard clinical process. Limitations are also balanced by the involvement and engagement of patients in the development of the educational video intervention and the focus on patients with type 2 diabetes, an important medical problem which is increasing in prevalence in the United States.

### 4.2 Innovation

Our novel methodological approach involved testing an educational video for patients that dispelled myths about provider-patient communication, provided examples of how to talk to your provider, used humor to engage the viewer, and was delivered in the weeks preceding the telehealth visit. Our patient-focused education video intervention was effective at improving patient engagement and therapeutic alliance with the provider.

### 4.3 Conclusion

The study had several qualities of pragmatic design including a population close to routine practice (in the VA) because there were few inclusion or exclusion criteria and because the study intervention was evaluated as part of routine clinical practice. Because of these pragmatic features health care systems may be able to implement this intervention with less cost than interventions requiring specially trained personnel. Given that many patients receive pre-visit reminders and instructions in current practice, the “Speak Up” video could be provided to patients prior to their visit as an online internet link by text message or email to allow pre-visit viewing on a desktop or laptop computer, handheld device or other internet connected device or alternatively for those without internet as a mailed DVD. The video could be included as part of orientation for new patients. Because the communication strategies demonstrated in the video could be useful for patients with other conditions the video could serve as a model for interventions in other medical and surgical conditions. While training patients to become more active participants in their medical visits is empowering and likely to generate positive outcomes as our results show, this should not minimize efforts to involve providers in quality communication. As such, the pamphlets for providers developed in this investigation may be a useful resource for providers preparing to conduct telehealth visits.

Our educational video was well-accepted by patients and was shown to be useful as pre-visit preparation for patients prior to a primary care telehealth visit. This study showed the efficacy of a pre-visit video to improve patient engagement and therapeutic alliance after video telehealth visits with their provider. Future studies should be conducted in larger groups to evaluate effectiveness and implementation of pre-visit videos that promote patients' active participatory communication. Future studies might also evaluate the use of pre-visit videos with virtual agents to help patients prepare for their visits and to practice active participation behaviors in a simulation with the virtual agent.

The following are the supplementary data related to this article.

Supplemental Fig. A.1 Pamphlet for Patients – Getting Ready for Your Telehealth Visit. Supplemental Fig. A.1

Supplemental Fig. A.2 Pamphlet for Providers – Making the Most of Your Patient's Video Visit – 5 Habits for Successful Communication. Supplemental Fig. A.2

## Declaration of Competing Interest

The authors declare the following financial interests/personal relationships which may be considered as potential competing interests:

Howard Gordon reports financial support was provided by US Department of Veterans Affairs.
