# Supplementary material for: A brief pre-visit educational video improved patient engagement after telehealth visits; results from a randomized controlled trial
Source: PEC Innov. 2022 Sep 5;1:100080. doi: 10.1016/j.pecinn.2022.100080 (PMC10194152; doi:10.1016/j.pecinn.2022.100080)

What is clinical video telehealth?

Telehealth uses video conferencing to securely provide you access to health care services from distant locations. Providers and patients who are in separate places can see each other and hear each other in real-time.

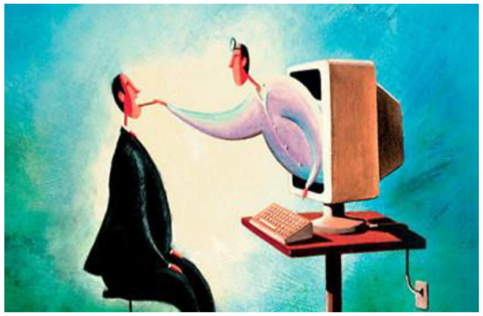

Resources for Telehealth

My HealtheVet- Explore ways to monitor and improve your health
https://www.myhealth.va.gov/mhv-portal-web/web/myhealthevet/how-to-use-mhv

Veterans health care library- Includes a variety of patient education material
http://www.veteranshealthlibrary.org

This brochure is developed as a part of an IRB approved study to improve doctor-patient communication in veterans, led by Dr. Howard S. Gordon and Dr. Ravi K. Gopal.

VA Central Institutional Review Board: 14-22: “Encouraging Patient-Centered Communication in Clinical Video Telehealth Visits”

If you have any questions about the validity of the study, you can contact the VA Central IRB at 1-877-254-3130 or study staff at 312-569-7339

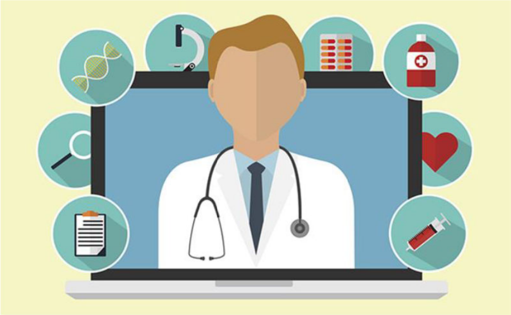

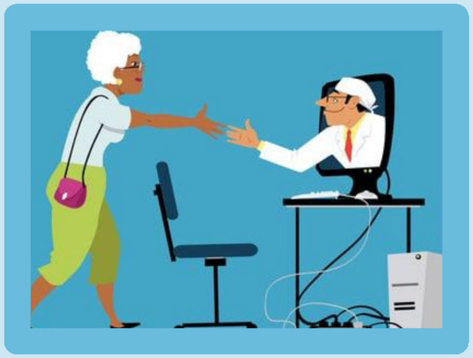

GETTING READY FOR YOUR TELEHEALTH VISIT

U.S. Department of Veterans Affairs

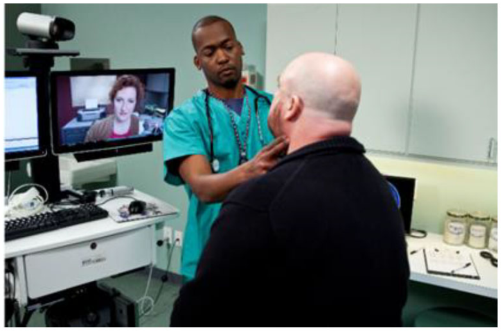

How does it work?

- The telehealth technician will make the connection so you will see the doctor and the doctor will see you
- The technician can use cameras and tools to do the physical exam

1 What should I do before my appointment?

- **Be prepared!**
  - Bring a list of three most important things for you to discuss with your doctor
  - Bring all your medications
  - Bring any health information you collected
    - Example: bring your glucometer
  - Bring a pen and paper to take notes
  - Bring hearing aid and reading glasses
  - Please come 15 minutes early

2 What should I do during my appointment?

- **Tell your doctor…**
  - What is most important to you
  - What problems or concerns you have
    - Example: "I'm feeling tingling in my feet."
- **Ask your doctor…**
  - All your questions! Don't be afraid the doctor will think your question is stupid
  - When there is something you don't understand
- **Make a request…**
  - If you need something
    - Example: "I need refills on my medication."
  - If you need to schedule another appointment to discuss more
- **SPEAK UP…**
  - When you cannot see or hear the doctor
  - When you think the doctor did not hear what you said
  - When the doctor cannot see a physical feature you want to show on the screen

- ***Remember…***
  - If the doctor is not looking at you, he or she may be looking at the chart
  - You can always ask if he or she heard what you said
  - The doctor may not see your body language

3 What should I do at the end of my appointment?

Review your plan with the doctor:

- Do you remember the plan?
- Did you get what you needed from your visit?
- Are you confident you can carry out the plan?
- Do you know when and how to schedule your follow up visit?

Contact your doctor or nurse if you need help or did not check all the boxes above

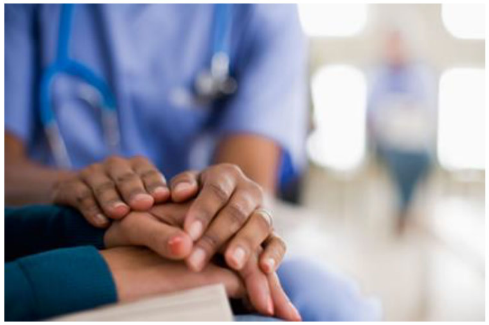

Supplement: Supplemental Fig. A.1 — Pamphlet for Patients – Getting Ready for Your Telehealth Visit. [file mmc1.pdf]
