# Supplementary material for: A brief pre-visit educational video improved patient engagement after telehealth visits; results from a randomized controlled trial
Source: PEC Innov. 2022 Sep 5;1:100080. doi: 10.1016/j.pecinn.2022.100080 (PMC10194152; doi:10.1016/j.pecinn.2022.100080)
Supplement: Supplemental Fig. A.2 — Pamphlet for Providers – Making the Most of Your Patient's Video Visit – 5 Habits for Successful Communication. [file mmc2.pdf]

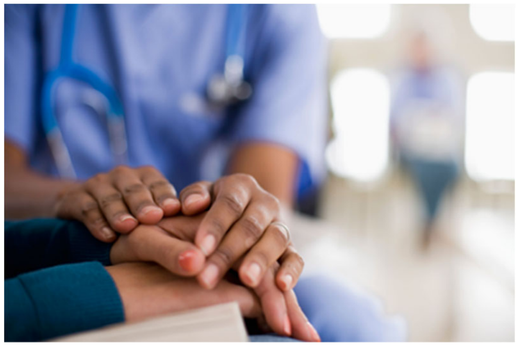

4 Demonstrate Empathy

Be open to patient’s emotions

- Assessing body language may be challenging
- Pay attention to voice tone and volume
- Take opportunities to respond empathetically

Be aware of your own reactions

- Focus on being present and mindful of your actions

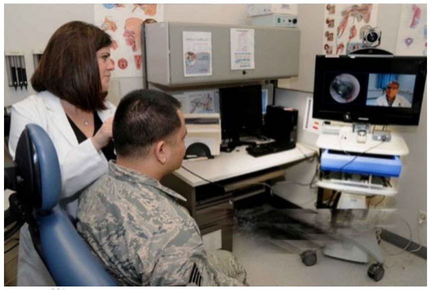

5 Invest in the End

Discuss assessment and plan

- Explain rationale for tests and treatment options
- Review risks, benefits, and alternatives

Involve patient in making decisions

- Explore goals and options, listening for the patient’s preferences
- Assess patient's ability and motivation to carry out plan

Complete the visit

- Ask for additional questions
  - "What questions do you have?"
- Assess satisfaction
  - "Did you get what you needed?"
- Provide patients with educational materials by mail or by asking technicians to print
- Reassure patient of ongoing care

Making the Most of Your Patient’s Video Visit

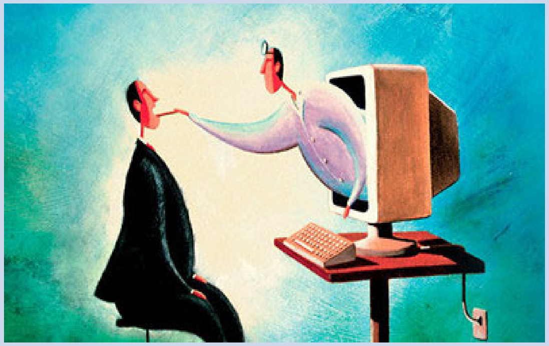

5 Habits for Successful Communication

VA HEALTH CARE Defining EXCELLENCE in the 21st Century

1 Prepare for the Visit

Plan Ahead

Get Ready

- Review the chart
- Know who to contact in case of a patient emergency
- Know how to contact the technician
- Know the remote site's capabilities
  - Example: Labs and pharmacies

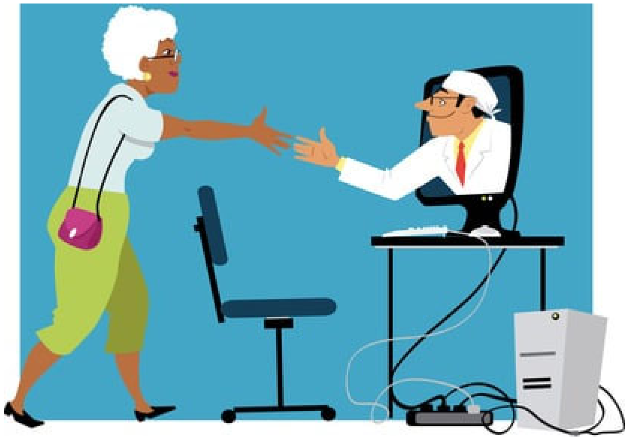

2 Invest in the Beginning

Create rapport quickly

- Look at the "web cam" to make eye contact
  - Check your self-view and ask if patient can see and hear you
  - Tell the patient that you may look away from camera to take notes during the visit
- Ask who is in the patient's room and greet them
- Address patient's privacy concerns
  - "Are you comfortable having people in your room during the visit?"
- Use verbal cues - patient may not be able to see your body language
- Convey knowledge of patient's history by commenting on prior visit or problem

Elicit Patient's concerns

- Start with open-ended questions
  - "What would you like help with today?"

Plan the visit

- Repeat concerns and requests back to check understanding
- Prioritize
  - "Let's make sure we talk about X and Y. If we don't have time for Z, we can schedule you for another appointment."
- Let patient know what to expect
  - "How about we start with talking about…, then the technician will help you do the exam and we'll go over possible tests/ways to treat this? Sound Ok?"

3 Elicit the Patient's Perspective

Ask for Patient's Ideas

- Make eye contact with patient
- Assess patient's point of view
  - "What do you think is causing your symptoms?"
  - "What worries you most about this problem?"

Elicit specific requests

- Ask about the patient's goal in seeking care
  - "When you've been thinking about this visit, how were you hoping I could help?"

Explore the impact on the patient's life

- Check context
  - "How has the illness affected your daily life?"

Resources:
- VHA Telehealth Services: http://vaww.telehealth.gov/
- VA Video Connect: http://vaww.telehealth.va.gov/pgm/vvc/index.asp

About this brochure:
- This brochure is developed as a part of an IRB approved study to improve doctor-patient communication in veterans, led by Dr. Howard S. Gordon and Dr. Ravi K. Gopal. If you have any questions about the validity of the study, you can contact VA Central IRB at 1-877-254-3130 or study staff at 312-569-7339.
- The content is adapted from: *Frankel RM, Stein T. Getting the most out of the Clinical Encounter: The Four Habits Model. The Permanente Journal 1999; 3(3):81.*
- VA Central Institutional Review Board: 14-22: "Encouraging Patient-Centered Communication in Clinical Video Telehealth **Visits**"
